# Management of Normothermic Regional Perfusion Performance in Uncontrolled Versus Controlled Donation After Circulatory Death: A Multi-Center Investigation

**DOI:** 10.3390/jcm14197053

**Published:** 2025-10-06

**Authors:** Chiara Lazzeri, Davide Ghinolfi, Manuela Bonizzoli, Daniele Cultrera, Paolo Lo Pane, Arianna Trizzino, Arianna Precisi o Procissi, Giuseppe Feltrin, Adriano Peris

**Affiliations:** 1 Regional Transplant Center, 50135 Florence, Italy; adriano.peris@gmail.com; 2 Division of Hepatic Surgery and Liver Transplantation, Azienda Ospedaliero Universitaria Pisana, 56126 Pisa, Italy; dghinolfi@yahoo.it (D.G.); arianna.trizzino@ao-pisa.toscana.it (A.T.); 3 ICU and ECMO Center, Azienda Ospedaliero Universitaria Careggi, 50134 Florence, Italy; bonizzolim@aou-careggi.toscana.it; 4 Coordinamento AV Centro, 50134 Florence, Italy; daniele.cultrera@uslcentro.toscana.it; 5 Coordinamento Locale, 50100 Livorno, Italy; paolo.lopane@uslnordovest.toscana.it; 6 Coordinamento Area Vasta Nord Ovest, 56121 Pisa, Italy; a.precisi@ao-pisa.toscana.it; 7 Italian National Transplant Center, 00162 Rome, Italy; giuseppe.feltrin@iss.it

**Keywords:** uncontrolled donation after circulatory death, controlled donation after circulatory death, normothermic regional perfusion, NRP management, NRP performance, lactate, liver transplant

## Abstract

**Introduction:** Controlled (c-) and uncontrolled (u-) DCDs are two entirely different types of donors, mainly because the duration of ischemic and reperfusion injury differs between them. We hypothesized that normothermic regional perfusion (NRP) management and performance (as indicated by the dynamic changes in blood flow and lactate) might be different in uDCDs and in cDCDs. **Methods:** We assessed 99 DCD donors that were consecutively evaluated by the Tuscany Regional Transplant Center from 2020 to 2024 (multi-center investigation), focusing on the comparison between NRP performance and management in uDCDs (n = 44) vs. cDCDs (n = 45). **Results:** NRP duration was significantly higher in uDCDs compared to cDCDs (*p* = 0.001). During NRP, we observed no changes in lactate values in uDCDs and cDCDs, a significant increase in transaminases, and a progressive reduction in NRP blood flow rates despite the administration of more fluids. Throughout the entire NRP duration, pH values were significantly lower and glucose levels were higher in uDCDs compared to cDCDs, even though a higher dosage of bicarbonate and insulin units were administered in uDCDs. **Conclusions:** In our series, we documented that NRP performance and management differed in uDCDs compared to cDCDs. This phenomenon may be mainly related to the different duration of the ischemic injury between these two types of donors. During NRP, uncontrolled DCDs showed a more severe metabolic derangement, which was only partially reversable by a more aggressive treatment (higher fluid volumes, insulin and bicarbonate dosages). Our results strongly suggest that there is likely space for optimization of NRP management in DCDs. Further research should address this issue, considering the disparity between the supply of organs and increasing transplantation needs.

## 1. Introduction

During donation after circulatory death (DCD), normothermic regional perfusion (NRP) allows the assessment of organ function following warm ischemic injury and changes the donor operation into a slower procedure rather than a super rapid recovery (SRR) [1].

Currently, NRP is mandatory in controlled DCD donors (cDCDs) in three European countries (Italy, France, and Norway) and is permitted in four (Spain, United Kingdom, the Netherlands, and Switzerland) [2]. In Italy, NRP is mandatory for both controlled and uncontrolled DCDs (uDCDs) because of the 20-min no-touch period required by Italian law for death certification with circulatory criteria. In Spain, between 2017 and 2019 [3], the number of liver transplants (LT) performed with NRP was higher than the number performed with SRR. Utilization of NRP is expected to increase in several countries in the next few years, including in the United States [4,5].

The main challenge with DCD donors is that ischemia–reperfusion injury mechanisms are not completely understood in either uDCDs or cDCDs. NRP has been reported to restore energy substrates that are depleted during warm ischemia and to reduce levels of nucleotide degradation products, thus helping to improve organ quality and maintenance before the subsequent cold ischemia. NRP has also been shown to induce the production of endogenous antioxidants. However, the exact mechanism by which NRP acts at a cellular level still needs to be fully explored [6,7].

Controlled and uncontrolled DCDs are two entirely different types of donors, mainly because the duration of ischemic and reperfusion injury differs between them. It is conceivable to suppose that NRP management and performance (as indicated by the dynamic changes in blood flow and lactates) might be different in uDCDs and cDCDs. To date, few data are available on the differences in NRP management between these two types of donors since most papers focus on the technical issues associated with NRP implantation, parameters for organ viability assessment, and transplant outcomes. Further, no data are provided on NRP management (i.e., fluids, bicarbonate, insulin) [8].

We aimed to assess NRP management in DCD donors that were consecutively assessed by the Tuscany Regional Transplant Center from 2020 to 2024, focusing on the comparison between uDCDs and cDCDs. Liver transplant outcomes from DCD donors were also assessed.

## 2. Methods

In our retrospective analysis of a prospective multi-center protocol, we assessed NRP performance and management in 99 DCDs consecutively evaluated by the Tuscany Regional Transplant Center from 2020 (August–December) to 2024, focusing on the comparison between uDCDs (n = 44) and cDCDs (n = 45). uDCDs are performed in two teaching hospitals (Careggi–Florence and Siena), while cDCDs are performed in Careggi and Pisa (teaching hospitals) and in seven peripheral hospitals.

The study was conducted according to the guidelines of the Declaration of Helsinki, and approved by the Ethics Committee of CEAVNO (Comitato Etico di Area Vasta Nord Ovest, Ethics Committee of the North West Area), with protocol code #17848 and approval on 23 July 2020” (Clinicaltrial.gov #NCT04744389).

### 2.1. uDCD Program—Bicompartimental Model [8,9]

This model can be applied in hospitals equipped with a local extracorporeal membrane oxygenation (ECMO) team that is available 24 h a day, 7 days a week.

In all cases of witnessed cardiac arrest, the emergency medical system alerts the physician in charge at the emergency department that either the therapeutic fast track or the donation path is required. A certified ECMO team (including an intensivist, a cardiac surgeon, a cardiologist, and a perfusionist) is immediately activated. Depending on the context, the procurement and transplant coordinator may also be mobilized. In the emergency department, in the case of irreversible circulatory death certification, local procurement coordination is involved. If all the inclusion criteria are fulfilled, the patient is recognized as a potential donor.

The inclusion criteria for a potential donor are the following: age of 18–65 years; witnessed cardiac arrest; clear patient identification; his/her relatives are present; no flow time <20 min; cardiac arrest to hospital arrival time <90 min; and a cardiac arrest to ECMO start time <150 min. According to Italian law, a 20-min no-touch period is currently required to declare death, along with a continuous electrocardiographic recording indicating the absence of any cardiac electrical activity. During the “no-touch period”, the family of the potential donor is informed of the death and of the possibility for organ donation. If the family is favorable to donation, NRP is started by placing the cannula (femoral artery) with a rapid Seldinger technique. The procedure of cannulation is performed under echocardiographic guidance. Supradiaphragmatic aortic occlusion is achieved with the placement of an aortic balloon. Transesophageal echocardiography is used to ensure that the thoracic aorta is totally occluded with the aortic occlusion balloon. A chest radiograph is obtained a few minutes later to document the aortic occlusion.

Donor management during NRP is performed by the local coordinator in collaboration with the Regional Center for Transplant Coordination, as previously described [10]. The NRP targets are mainly represented by the maintenance of NRP flows >2 L/min. Any acid–base imbalance, electrolyte abnormalities, and hypo- or hyperglycemia are also treated. Blood samples from the ECMO device are obtained just after starting NRP, after 2 and 4 h (NRP1, NRP2, and NRP3), respectively, for the measurements of serum lactate levels, transaminases (alanine transaminase, ALT; aspartate transaminases, AST), and blood flow.

Organ retrieval is scheduled 4 h from NRP start, unless NRP instability occurs.

Donor evaluation and donor risk assessment are performed in this time window (from death certification to organ retrieval) by the local transplant coordinator (donor evaluation) and the Regional Transplant Center (donor evaluation and risk assessment).

### 2.2. cDCD Program

In the Tuscany Region, the cDCD was implemented in 2018. In 2021, the Regional Transplant Authority launched a cDCD NRP mobile program to make the cDCD pathway feasible, even in peripheral hospitals.

The availability of an NRP mobile team was pivotal to achieving the implementation of cDCD programs, especially in peripheral hospitals that were not equipped with a local ECMO team. In situations where there was a lack of guidelines and/or recommendations on the characteristics of an NRP mobile team, the Regional Transplant Authority opted to “convert” existing ECMO mobile teams into “NRP mobile teams” for cDCD implementation in peripheral hospitals. This decision was supported by the need to guarantee and achieve the lowest incidence of complications and the shortest warm ischemic time during the cDCD process. Complications during NRP implantation would make the donor unsuitable for transplantation and a long warm ischemia time is known to affect graft outcome [2].

According to the National Transplant Guidelines, whenever a potential cDCD is identified in any of the intensive care units in the Tuscany Region, the local procurement coordinator is activated. Thereafter, the suitability of the potential cDCD for organ transplantation is assessed by the Regional Transplant Center in collaboration with the local procurement coordinator. Whenever a cDCD is deemed suitable for organ donation, the NRP mobile team is activated. According to the regional cDCD pathway, the procurement coordinator activates the nearest NRP mobile team, in accordance with the Regional Transplant Center.

In all cDCDs, a total body angio CT scan is performed together with an ultrasound evaluation of the groin bifemoral artery and vein to assess suitability for NRP implantation.

Warm ischemia time (WIT) is defined as the time from the withdrawal of life support to NRP start.

### 2.3. Normothermic Regional Perfusion Monitoring and Management [10]

NRP is started after percutaneous cannulation of the femoral vessels with a rapid Seldinger technique, as previously described [10]. Supra-diaphragmatic aortic occlusion is achieved with the placement of an aortic balloon whose correct position is confirmed by transesophageal echocardiography and, afterwards, by chest X-ray. The responsibilities of the mobile NRP team are the cannulation process and the maintenance of an efficacious NRP. A strict collaboration between the NRP mobile team and the surgical procurement team is mandatory for the success of the entire procedure. Heparin is administered at a dosage of 20.000 IU once the donor systolic blood pressure drops below 50 mmHg or oxygen saturation drops below 70%.

Each procurement center in the Tuscany Region implemented local protocols for DCD donation, including the intervention of the NRP mobile team.

According to our protocols, the goals during NRP management are as follows:

- NRP flows (median) > 2 L/min;
- pH ≥ 7.4;
- Glucose (median) ≤ 200 mg/dL.

Glucose management is performed by rapid insulin administration (repeated boluses) and fluid management is performed by ringer lactate administration. Red blood cells are administered to maintain Hb in the range of 8–10 g/dL. Acidosis is corrected by bicarbonate administration (repeated boluses). In uDCDs, no vasoactive is administered. In cDCDs, vasoactive drugs are administered if necessary (at the discretion of the local transplant coordinator).

### 2.4. Liver Viability Assessment During NRP

Liver grafts were considered suitable for procurement if the following parameters during NRP were satisfied [8,9,10,11,12,13]:

- ALT < 1000 UI/L 4 h after commencing NRP;
- Stable or downward trend in serum lactate concentration;
- NRP flows (median) > 2 L/min.

At the time of procurement, the liver was evaluated for its macroscopic appearance. A liver biopsy was mandatory and a graft was discarded if the following conditions were met:

- Macrovescicular steatosis > 40%;
- Necrosis > 10%;
- Fibrosis > 2.

All liver grafts were eventually perfused ex situ in hypo- or normothermic perfusion based on NRP performance [12]. Early liver dysfunction and 30-d graft survival were also assessed. The utilization rate was reported as the ratio between utilized livers and retrieved livers.

### 2.5. Statistical Analysis

Data were processed with the IBM-SPSS 20 statistical package (SPSS Inc., Chicago, IL, USA). Categorical variables were reported as frequencies and percentages. Continuous variables were reported as mean ± standard deviation (SD) or median (range, min–max), as needed. Comparisons between the groups were performed using the chi-square test for categorical data. Student’s *t*-test and the Kruskal–Wallis test were performed for continuous data. A *p* value less than 5% was interpreted as statistically significant.

## 3. Results

The study population, shown in Table 1, comprises 45 cDCDs and 44 uDCDs. Considering age-related inclusion criteria, uDCDs were younger than cDCDs (*p* = 0.0001). Males were prevalent in uDCDs (*p* = 0.023). NRP duration was significantly higher in uDCDs compared to cDCDs (*p* = 0.001).

### 3.1. NRP—uDCD

As depicted in Table 2, during NRP, lactate values did not show significant changes, while a progressive and significant increase was observed both in ALT and AST values, respectively. NRP blood flow rates exhibited a progressive reduction from start to organ retrieval. A significant progressive increase was observed in pH values and glucose levels, respectively (*p* < 0.001 for both).

### 3.2. NRP-cDCDs

Table 2 shows the results of a dynamic assessment during NRP in cDCDs. A progressive and significant reduction in lactate values was observed (*p* < 0.001), while AST, ALT, and blood values did not change during NRP. A significant progressive increase was observed in pH values, but glucose levels decreased (*p* < 0.001 for both).

### 3.3. Dynamic Assessment During NRP—uDCDs vs. cDCDs

Table 3 shows the comparison between uDCDs and cDCDs regarding NRP parameters and management. Lactate values were significantly higher throughout the whole NRP duration in uDCDs compared to cDCDs. Similarly, ALT and AST values were higher in uDCDs than in cDCDs. Conversely, NRP blood flow was lower in uDCDs than in cDCDs throughout the entire NRP duration, despite the administration of more fluids in uDCDs (Table 4). Throughout the entire NRP duration, pH values were significantly lower and glucose levels were higher in uDCDs than in cDCDs, even though a higher dosage of bicarbonate and insulin units were administered in uDCDs. Norepinephrine was administered in only one cDCD at a low dosage (0.05 mcg/kg/min).

### 3.4. Liver Transplants and Outcomes

Among the 45 cDCDs, six livers were not transplanted due to NRP and machine perfusion parameters. Thirty-nine liver transplants were performed with a utilization rate of 87%. EAD was observed in eight recipients (8/39, 20%). Four recipients died: acute myocardial infarction occurred in one case and septic shock occurred in the remaining three cases. Among the dead recipients, graft liver dysfunction was present in one. At the 30-d follow-up, liver graft function was present in all but one recipient (97.4%).

Among the uDCDs, 16 livers were transplanted, with a utilization rate of 36% (16/44). EAD was observed in six recipients (7/16, 43%). Five recipients died because of septic shock. Among the dead recipients, graft liver dysfunction was present in four. At the 30-d follow-up, liver graft function was present in all but four recipients (75%).

## 4. Discussion

The main finding of the present investigation, which was performed in 99 DCD donors (45 cDCDs and 44 uDCDs), is that NRP performance and management are different in uDCDs compared to cDCDs.

Considering the lack of accepted global targets for NRP management in DCD donors, the novelty of the present investigation is, in our opinion, the assessment of NRP performance and management in uDCDs and cDCDs. In a small series of 18 DCDs (11 cDCDs and 7 uDCDs), a more severe metabolic derangement was observed in uDCDs during NRP. However, no information was provided on NRP management in this study [14]. In a larger series of DCD donors, we confirmed a more severe metabolic imbalance in uDCDs. However, we also observed that this metabolic derangement was partially reversed at the end of NRP in uDCDs despite more aggressive treatments, as indicated by higher fluid volumes, insulin boluses, and bicarbonate dosages administered.

These findings may be mainly related to the different duration of the ischemic injury between these two types of donors (150 min in uDCDs versus about 40 min in cDCDs) [2,8].

In uDCDs, the ischemic–reperfusion injury comes from an “ischemic time frame” of no-flow/low ischemia (which may be up to 150 min, that is, from cardiac arrest to NRP start) and an “ischemic–reperfusion time frame” (that is, after NRP start) that lasts up to four hours. All uDCDs suffered from refractory cardiac arrest. In these donors, the ischemia reperfusion injury shares the pathophysiological mechanism(s) described in the so-called *post-cardiac syndrome* (PCAS), specifically in its early post-arrest phase (20 min to 6 h) [15,16]. This syndrome is a complex occurrence characterized by a constellation of events that worsen over time. The higher lactate values and the increasing values of transaminases and glucose observed in uDCDs rather than cDCDs may support this contention. Various triggers contributing to post-cardiac syndrome have been described, such as impairment of mitochondrial oxidative phosphorylation (due to oxygen deprivation), acid–base imbalance (causing cellular dysfunction), depletion of energy stores resulting in cell edema, cell influx, and increased production of reactive oxygen species. All these tissue injuries trigger the activation of an inflammatory cascade, whose impact is directly proportional to the duration of the ischemia. In keeping with these contentions, our group recently reported that, during NRP, higher plasma levels of inflammatory and liver damage markers (including α-glutathione s-transferase, sorbitol-dehydrogenase, malate dehydrogenase 1, liver-type arginase-1, and keratin-18) were observed in uDCDs compared to cDCDs [13]. The significant reduction in NRP blood flow in uDCDs may be related to progressive cell leakage and edema formation (similarly to post-cardiac syndrome [15,16]), which may lead to the development of an abdominal compartmental syndrome over time. In the single uDCD donor, the reduction in blood flow during NRP accelerated organ retrieval at our center, since low (<1.5 L/min) blood flow may impair abdominal organ function. This cascade of events is only partially reversed by aggressive treatment. The latter finding might suggest the need to reduce NRP duration to 2–3 h in uDCDs, but further research is needed to understand the effects of this modified protocol. On the other hand, the ischemic time frame of 150 min can be hardly reduced considering the median time from cardiac event and hospital arrival (80 min), and the 20-min no-touch period required by Italian law for death certification. The more severe ischemic reperfusion injury observed in uDCDs could contribute to the worse outcomes observed following liver transplants in uDCDs vs. cDCDs. However, in uDCDs, recent evidence documents that a proper donor and recipient selection are pivotal factors in achieving better outcomes in these donors [17,18]. In our experience, uDCDs should be further used in patients with limited portal hypertension, good performance status, and advanced HCC exceeding Milan criteria.

Conversely, in cDCDs the brief duration of ischemia (about 40 min) followed by reperfusion might resemble “remote ischemic preconditioning before the ischemic event” [17,18]. This hypothesis is supported by the lower levels of transaminases, glucose, and lactates observed in our study during NRP in cDCDs (compared to uDCDs), indicating a lower degree of ischemic–reperfusion injury. Similarly, lower plasma levels of inflammatory and liver damage markers were observed in cDCDs during NRP (compared to uDCDs) [13]. The protective effect of ischemic preconditioning followed by reperfusion was confirmed by experimental models [19,20,21,22]. In our series, treatment (fluid replacement, insulin, and bicarbonate) managed to normalize pH and metabolic imbalance.

Further research is needed to identify the optimal treatment during NRP, especially regarding vasoactive use.

In our experience, the sequential use of NRP and MP has improved organ utilization rate and health outcomes. The use of normothermic machine perfusion should be mandatory in any situation where NRP is borderline acceptable or when a re-evaluation should be performed.

### Limitation of the Study

This is an observational study that includes a small population of DCD donors. In our series, 44 uDCDs were included. However, the uDCD program is active in a limited number of countries and donation centers. The 20-min no-touch period requested by Italian law for death certification makes our results not easily transferable to other countries. We acknowledge that the liver transplant outcome is also affected by the use of ex situ machine perfusion; however, we did not address this issue since it is outside the aim of our investigation.

## 5. Conclusions

In our series, we documented that NRP performance and management differed in uDCDs compared to cDCDs. This phenomenon may be mainly related to the different duration of the ischemic injury between these two types of donors. During NRP, uncontrolled DCDs showed a more severe metabolic derangement, which was only partially reversable by a more aggressive treatment (higher fluid volumes, insulin and bicarbonate dosages). Our results strongly suggest that there is likely space for the optimization of NRP management in DCDs. Further research should address this issue, considering the disparity between the supply of organs and increasing transplantation needs.

## Figures and Tables

**Table 1:** Study population.

	cDCD	uDCD	*p*
Number	45	44	
Age (yrs, mean ± SD)	68 ± 15	52 ± 9	0.0001 *
Gender (M/F, n., %)	31/14, 69/31%	39/5, 89/11%	0.023 #
BMI (kg/m^2^, mean ± SD)	30.4 ± 4	29.7 ± 7	0.467 *
Cause of death (n., %)			
Cardiac arrest		44 (100%)	
Stroke	12 (27%)		
Post-anoxic encephalopathy	31 (69%)		
Trauma	2 (4%)		
Cardiac arrest—NRP run—WIT (median, range, min–max)		145.5 (60–175)	
NRP duration (hour, median range, min–max)	4 (2–6)	6 (4–9)	0.001 &

uDCD, uncontrolled donation after circulatory death; cDCD, controlled donation after circulatory death; M, males; F, females; SD, standard deviation; BMI, body mass index; WIT, warm ischemia time; NRP, normothermic regional perfusion; *, Student’s *t*-test; #, chi-square test; &, ANOVA test.

**Table 2:** Dynamic assessment during NRP in uDCDs and cDCDs.

**uDCD**				
**Median (range, min–max)**	**NRP1**	**NRP2**	**NRP3**	***p***
Lactate (mmol/L)	18 (8–29)	20 (13–27)	18.5 (8–30)	0.126 &
AST (UI/L)	223.5 (61–2375)	287 (88–2243)	384 (56–2900)	0.045 &
ALT (UI/L)	237 (89–2377)	286.5 (65–2071)	395.5 (84–2125)	0.013 &
Blood flow (L/min)	3 (1–4)	3 (1–4.3)	2 (1–4)	0.001 &
pH	6.86 (6.17–7.33)	7.2 (6.17–7.50)	7.3 (6.7–7.49)	<0.001
Glucose (mg/dL)	359 (105–580)	276 (85–461)	198 (46–451)	<0.001
**cDCD**				
**Median (range, min–max)**	**NRP1**	**NRP2**	**NRP3**	
Lactate (mmol/L)	9 (6.9–13.3)	7 (3.4–11.3)	3 (2–10.1)	<0.001 &
AST (UI/L)	43 (11–1221)	56 (12–1736)	62 (11–2054)	0.305 &
ALT (UI/L)	43 (12–1340)	50 (12–1500)	61 (16–1664)	0.375 &
Blood flow (L/min)	3 (2.41–4.3)	3 (2.3–4.15)	3 (2.5–4.2)	0.810 &
pH	7.09 (6.72–7.47)	7.4 (7.19–7.57)	7.45 (7.30–7.58)	<0.001 &
Glucose (mg/dL)	282 (144–405)	232 (102–385)	168 (97–380)	<0.001 &

uDCD, uncontrolled donation after circulatory death; cDCD, controlled donation after circulatory death; AST, aspartate transaminase; ALT, alanine transaminase; &, ANOVA test.

**Table 3:** Comparison of NRP dynamic assessment between uDCDs and cDCDs.

Median (Range, Min–Max)	uDCD	cDCD	
Lactate (mmol/L)			
1	18 (8–29)	9 (6.9–13.3)	<0.0001 &
2	20 (13–27)	7 (3.4–11.3)	<0.0001 &
3	18.5 (8–30)	3 (2–10.1)	<0.0001 &
AST (UI/L)			
1	223.5 (61–2375)	43 (11–1221)	0.001 &
2	287 (88–2243)	56 (12–1736)	<0.0001 &
3	384 (56–2900)	62 (11–2054)	<0.001 &
ALT (UI/L)			
1	237 (89–2377)	43 (12–1340)	0.005 &
2	286.5 (65–2071)	50 (12–1500)	<0.0001 &
3	395.5 (84–2125)	61 (16–1664)	<0.0001 &
Blood flow (L/min)			
1	3 (1–4)	3 (2.41–4.3)	<0.0001 &
2	3 (1–4.3)	3 (2.3–4.15)	<0.0001 &
3	2 (1–4)	3 (2.5–4.2)	<0.0001 &
pH			
1	6.86 (6.17–7.33)	7.09 (6.72–7.47)	<0.0001 &
2	7.2 (6.17–7.50)	7.4 (7.19–7.57)	<0.0001 &
3	7.3 (6.7–7.49)	7.45 (7.30–7.58)	<0.0001 &
Glucose (mg/dL)			
1	359 (105–580)	282 (144–405)	0.01 &
2	276 (85–461)	232 (102–385)	0.05 &
3	198 (46–451)	168 (97–380)	0.02 &

uDCD, uncontrolled donation after circulatory death; cDCD, controlled donation after circulatory death; AST, aspartate transaminase; ALT, alanine transaminase; &, ANOVA test.

**Table 4:** NRP management: comparison between uDCD and cDCD.

Median (Range, Min–Max)	uDCD	cDCD	
Ringer lactate (mL)	3500 (2000–5500)	600 (400–3200)	<0.0001 &
Packed red blood cell units (n.)	3 (2–5)	3 (1–6) n.45	0.76 &
Insulin therapy (units)	15 (5–35)	8 (5–10)	<0.0001 &
Bicarbonate (mEq)	600 (150–800)	100 (100–300)	<0.001 &

&, ANOVA test.

## Data Availability

The original contributions presented in this study are included in the article. Further inquiries can be directed to the corresponding author.
